# Older Age Is Associated With Confirmation Bias in Climate-Related Decision Making

**DOI:** 10.1093/geroni/igaf122.1399

**Published:** 2025-12-31

**Authors:** Julia Nolte, Yochai Shavit, Wee Qin Ng

**Affiliations:** Tilburg University, Tilburg, Noord-Brabant, Netherlands; Stanford University, Stanford, California, United States; Nanyang Technological University, Singapore, Singapore

## Abstract

Prior findings suggest that older adults are more likely to underuse and avoid negative or upsetting information. Yet, the extent to which they demonstrate higher levels of “confirmation bias”- the preference to engage with or assign more meaning to belief-consistent information and options - is still unclear. An online experiment examined the above research gap in the context of climate change, a divisive topic among older adults. A US adult lifespan sample (N = 206, 19–88 years, M = 51.20, SD = 17.77, 52% women, 28% Non-Hispanic White) engaged with information about three pro- and three anti-environmental fictional candidates running for a US office. Individual differences in confirmation bias were indexed as: (1) searching for more belief-consistent than -inconsistent information, (2) ranking candidates with belief-consistent campaigns more highly, and (3) perceiving belief-consistent information as more persuasive. In addition, participants responded to assessments of their sociodemographic background, goals, physical and mental health, affect, personality, cognition, and knowledge about climate change. In pre-registered analyses, we found evidence for confirmation bias in all three bias indices, which was positively associated with older age in the contexts of information search (p = .018) and candidate rankings (p = .016). Of all covariates considered, only older adults’ relatively better mental health accounted for older adults’ more biased information search behavior (but not their ranking decisions). Together, findings suggest that older adults may be more susceptible to disregarding belief-inconsistent information or options when making climate change-related decisions, and that this bias may serve emotional well-being goals.

